# Olfactory neuroblastoma in a goat

**DOI:** 10.1007/s11259-026-11505-9

**Published:** 2026-09-23

**Authors:** Sofía Bosco Mascaro, Alejandro Suárez-Bonnet, Bettina Dunkel, Sonja Jeckel, Bernat Martí-Garcia

**Affiliations:** 1 Departament de Sanitat i Anatomia Animals, Facultat de Veterinària, Universitat Autònoma de Barcelona (UAB), Campus de la UAB, Bellaterra, 08193 Catalonia Spain; 2 Pathobiology & Population Sciences, Royal Veterinary College, Hawkshead Lane, North Mymms, Hatfield, Hatfield, Hertfordshire AL9 7TA UK; 3 Department of Clinical Science and Services, Royal Veterinary College, University of London, London, UK

**Keywords:** Nasal, Olfactory neuroblastoma, Goat

## Abstract

Nasal tumours are uncommon in goats, with lymphoma, enzootic intranasal tumours and polyps being the most commonly reported. Nasal neuroectodermal tumours are rarely described and olfactory neuroblastoma (ONB), an infrequent neuroepithelial neoplasm originating from the cribriform plate, is poorly documented in this species. Herein we report the clinical presentation and necropsy findings of an 8-year-old goat with a confirmed diagnosis of ONB. The goat was presented in respiratory distress following a history of chronic respiratory noise and an intranasal, right-side infiltrative mass was detected on radiographs. Due to the poor prognosis, euthanasia was performed. Post-mortem examination revealed that the nasal cavity was bilaterally occupied by a round, creamy-grey, soft mass measuring 1 × 3 cm and extending to the nasopharynx. Histologically, a malignant neuroepithelial neoplasm was diagnosed based on its infiltrative growth and the characteristic pattern of Homer Wright and Flexner-Wistersteiner-like rosettes. Immunohistochemical characterization was performed using an antibody panel composed of cytokeratin AE1/AE3, cytokeratin 8, beta III tubulin, vimentin, COX-2, E-cadherin, chromogranin A and synaptophysin. The immunoprofile confirmed the diagnosis of an ONB, with neoplastic cells being only positive for cytokeratin AE1/AE3 and beta-III tubulin. To the best of the authors’ knowledge this is the first description of ONB in goats.

## Background

Neoplasms are uncommon in small ruminants, and only a limited number of reported tumour cases involve goats. Nasal tumours, included in the respiratory system, are scarcely reported in goats and when present, epithelial tumours are the most common, followed by lymphomas (Maxie 2025); (Vasconcelos et al. 2023). Clinical signs of nasal tumours include serosanguinous or mucopurulent nasal discharge, sneezing, inspiratory noise increased respiratory effort, and ocular discharge. Advanced cases tend to cause facial deformity and exophthalmos. Differential diagnoses include rhinitis, tooth root abscesses or osteomyelitis (Lubojemska et al. 2016). Neuroepithelial and neuroendocrine tumours are exceedingly rare in veterinary species, and especially uncommon in ruminants, highlighting the need for detailed clinical descriptions and comprehensive pathological characterisation of these neoplasms.

The aim of this case report is to describe the clinical, imaging, pathological, and immunohistochemical features of a nasal mass in an adult goat, ultimately diagnosed as a poorly differentiated olfactory neuroblastoma (ONB). To the authors’ knowledge, this represents the first documented case of ONB in a goat, contributing novel information to the veterinary literature.

## Case presentation

**Fig. 1:**
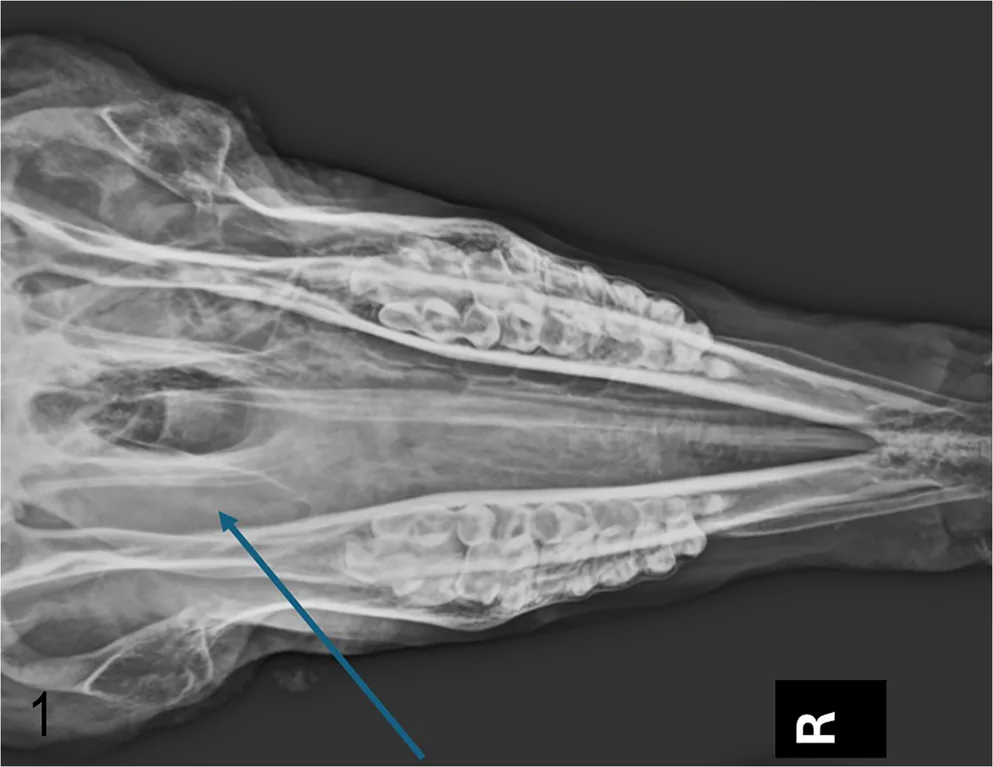
Dorso-ventral radiograph of the head showing mild deviation of the nasal septum to the left and a diffuse, heterogenous soft tissue opacity (blue arrow) within the right (R) nasal and paranasal sinus system, extending caudally to involve the cornual sinus. Loss of definition of the bone structures within the right sinus system is also appreciated

An 8-year-old, male pet goat was presented to the Equine Referral hospital of the Royal Veterinary College (RVC) with acute respiratory distress following an extended history of an inspiratory noise that had not responded to treatment with antimicrobials and corticosteroids. At admission, the animal was in respiratory distress with open mouth breathing, tachypnoea, an increased inspiratory and expiratory effort and loud inspiratory and expiratory noises. Bilateral mucohaemorrhagic nasal discharge was present and airflow was significantly reduced from both nostrils necessitating an emergency tracheostomy to allow further investigation. Radiographs of the head (Fig. 1) revealed an extensive soft tissue opacity withing the right nasal cavity; no radiographic changes were observed in the thorax. Due to the poor prognosis with no realistic treatment options, humane euthanasia was elected.

**Fig. 2:**
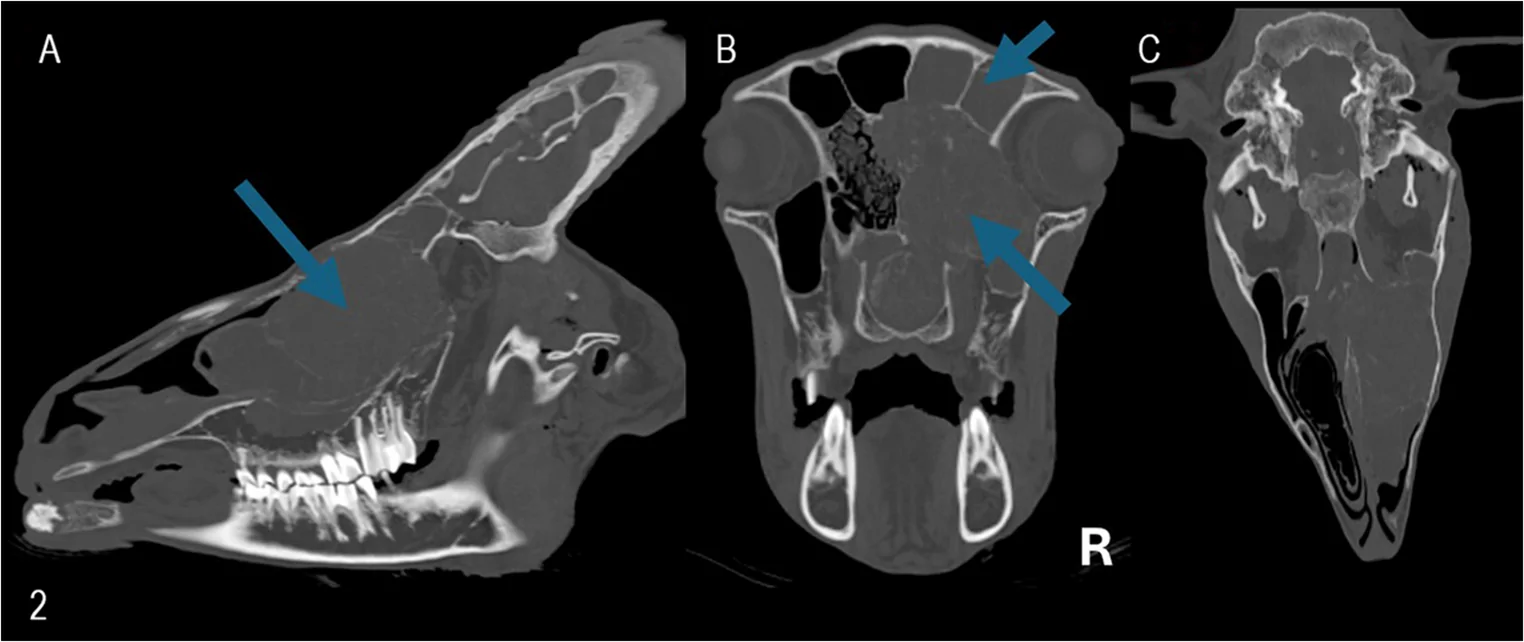
Computed tomography (CT) images of the head obtained postmortem showing the extend of the soft tissue mass, indicated by blue arrows, in a sagittal (A), transverse (B) and dorsal plane (C). The mass reaches from the nasal cavity and sinus systems into the nasopharynx and right cornual sinus

Post-mortem computed tomography (CT; Fig. 2) confirmed the presence of a large, infiltrative soft tissue mass occupying the entire right nasal cavity and sinuses, extending into the left nasal passages and the right horn.

**Fig. 3:**
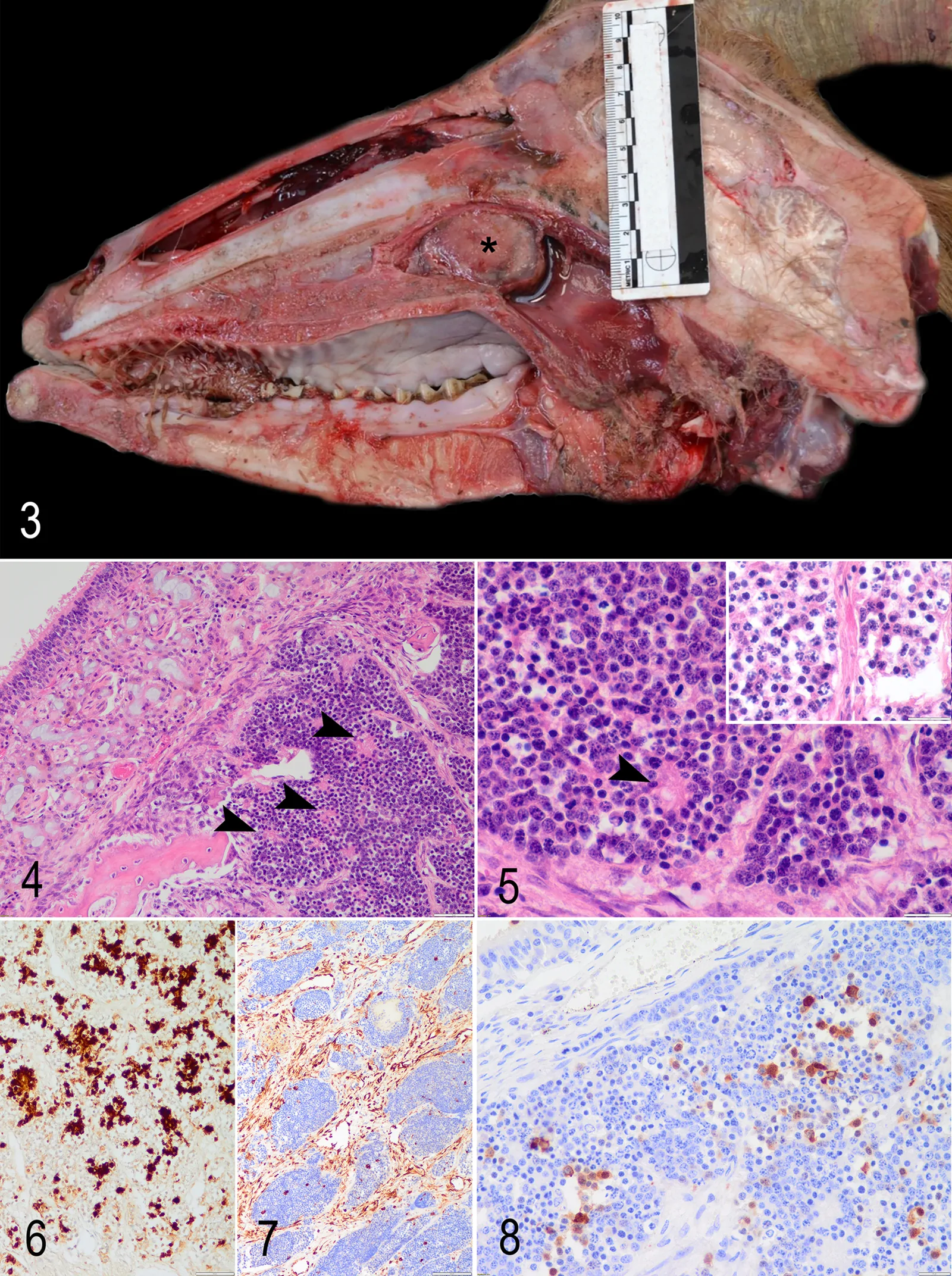
Right nasal cavity. Soft-tissue mass at the caudal aspect of the nasal cavity, associated haemorrhage and minimal invasion of the nasopharynx (asterisk). Photomicrograph of the nasal mass (sub-gross). The mucosa is effaced by a multi-lobular neoplasm forming anastomosing nests or sheets forming rosette-like structures (arrowheads). In the lower left side of the image, there is peri-osseous proliferation of immature fibroblasts, fragmentation of bony trabeculae (osteolysis) and increased numbers of active osteoblasts and osteoclasts (bone remodelling). Scale bar = 50 µm. Photomicrograph of the nasal mass (cellular detail). Cells are forming rosettes (arrowhead), polygonal, with scant amount of eosinophilic cytoplasm and poorly defined cytoplasmic borders. Nuclei are round, hyperchromatic, frequently fragmented (inset) and parabasal. Occasional mitotic figures are observed and anisocytosis and anisokaryosis are moderate. Scale bar = 20 µm. Neoplastic cells are consistently vimentin negative. Only stromal and rare inflammatory cells are vimentin positive. Scale bar = 50 µm. Scattered neoplastic cells are strongly positive for beta-III tubulin. Scale bar = 20 µm

The animal was submitted to the Pathology and Diagnostic Laboratories of the RVC for a post-mortem examination (PME) as per standard local protocol. The goat was in a fair body condition (2.5/5), and upon opening the nasal cavity there was a bilateral, round to ellipsoid, 1 × 3 cm cream-grey coloured, soft mass in the caudal aspect. This mass focally effaced the oblique transverse interfrontal septum in the left side and reached the frontal sinus of the right side, where it was associated with extensive intranasal hemorrhage (hematoma). This mass also breached the nasal septum and extended into the nasopharynx (Fig. 3). All the observed masses were associated with mucoid to haemorrhagic discharge.

Multiple tissue sections from the intra-nasal and pharyngeal mass were collected for histopathological evaluation and fixed in 10% neutral buffered formalin, routinely processed, sectioned at 4 μm, stained with haematoxylin and eosin and examined with a light microscope. Microscopic examination revealed a multi-lobular neoplasm forming anastomosing nests or sheets supported by a dense fibrovascular stroma. The neoplastic cells were often palisading around a moderately eosinophilic, amorphous matrix (rosette-like patterns) or around capillaries (pseudorosettes) (Rissi and Miller 2024). The cells were polygonal, with scant amount of cytoplasm and poorly defined cytoplasmic borders. Nuclei were round, hyperchromatic and parabasal, with marked nuclear fragmentation. Anisocytosis and anisokaryosis were moderate. Mitotic figures were sparsely observed across the tumor, with 15 mitotic figures observed in 2.37 mm^2^ (Figs. 3 and 3). In some areas near the trabecular bone there was a marked interstitial proliferation of immature fibroblasts (scirrhous response), fragmentation of bony trabeculae (osteolysis) and evidence of increased numbers of active osteoblasts and osteoclasts (bone remodelling). Histologically, a diagnosis of a likely neuroendocrine or neuroepithelial tumour was made and immunohistochemistry (IHC) using formalin-fixed paraffin-embedded serial tissue sections was performed using a broad antibody panel, including cytokeratin AE1/AE3, cytokeratin 8, E-cadherin, beta-III tubulin, chromogranin A, COX-2, vimentin and synaptophysin (Table 1), to determine the phenotype of the neoplastic cells. Canine tissues, including kidney, adrenal gland, brain and skin were used as positive controls. Negative controls were produced by replacing the primary antibody with phosphate-buffered saline solution. Immunohistochemically, neoplastic cells were positive for cytokeratin AE1/AE3 and beta III-tubulin only (Figs. 3, 3 and 3). Based on both the morphologic and immunohistochemical features, an olfactory neuroblastoma (ONB) was diagnosed.

**Table 1:** Summary of immunohistochemistry methodology

Antibody	Source	Host	Type	Clone	Antigen Retrieval	Dilution
AE1/AE3	Dako	Mouse	Monoclonal	AE1 AE3	Citrate buffer	1 in 100
Vimentin	Dako	Mouse	Monoclonal	V9	Citrate buffer	1 in 100
CgA	Dako	Mouse	Monoclonal	DAK-A3	Tris-EDTA buffer	1 in 100
Syp	Leica	Mouse	Monoclonal	27G12	Citrate buffer	Ready to use
III beta-tubulin	ThermoFisher	Mouse	Monoclonal	2G10	Citrate buffer	1 in 100
COX-2	ThermoFisher	Mouse	Monoclonal	SP21	Citrate buffer	1 in 50
EMA	ThermoFisher	Mouse	Monoclonal	E29	Citrate buffer	1 in 100
CK8	Leica	Mouse	Monoclonal	TS1	Citrate buffer	1 in 100

## Discussion

Neoplasms in goats are relatively rare, and nasal tumours are particularly uncommon. Goats account for approximately 10% of reported neoplastic cases in small ruminants, with the integumentary system being the most frequently affected (Vasconcelos et al. 2023).

Based on the animal’s age, clinical signs (Namjou et al. 2018);(Jahns and Cousens 2020) and localisation, nasal adenocarcinoma, either spontaneous or caused by the enzootic nasal tumour virus type 2 (ENTV 2) (Namjou et al. 2018);(Jahns and Cousens 2020) should be considered as the primary differential diagnosis. Other differential diagnoses include nasal polyps, squamous cell carcinoma and lymphoma(Maxie 2025);(Vasconcelos et al. 2023);(Li et al. 2025), but neuroendocrine/neuroepithelial tumours would be considered very unlikely.

ONB is an extremely rare neoplasia (Maxie 2025);(Vasconcelos et al. 2023);(Lubojemska et al. 2016). Macroscopically, it grows invasively and extends to the contralateral nasal cavity, causing extensive destruction of adjacent bony structures (Löhr 2013). The neoplastic cells are presumed to originate from neuroepithelial precursor cells (Pickuth et al. 1999) and arise within the upper nasal cavity at the level of the cribriform plate (Siudak et al. 2015). Only a few cases have been described in dogs and cats (Siudak et al. 2015);(Parker et al. 2010); (Martí-García et al. 2023), cattle (Anderson and Cordy 1981) and horses (Yamate et al. 2006). Olfactory neuroepithelial cells exhibit a high degree of pluripotential differentiation into neurogenic cell types, accounting for the tumour’s neuroectodermal characteristics (Martí-García et al. 2023); (Meuten 2017).

Histologically, ONBs grow in circumscribed lobules forming pseudorosettes and rosettes, often associated with an extracellular neurofibrillary matrix (Rissi and Miller 2024).

The microscopic differential diagnoses due to their potential histological similarities include neuroendocrine carcinomas (‘carcinoids’); non-keratinizing squamous cell carcinoma, sinonasal mucosal malignant melanoma and rhabdomyosarcoma (Lubojemska et al. 2016).

In the presented case, neuroepithelial differentiation of the tumour was suspected based on the microscopic morphology, and this was confirmed by immunohistochemical staining (Meuten 2017);(Vengušt et al. 2025);(Ninomiya et al. 2008). The histological distinction between carcinoids and neuroepithelial nasal tumours must be supported by differential expression of neuronal, epithelial and endocrine markers(Meuten 2017) (Table 2). Beta III-tubulin showed scattered positive immunoreactivity, supporting the neuroectodermal origin of the tumour and its neuroepithelial differentiation (Church et al. 2019). The negativity for neuroendocrine markers like chromogranin A and synaptophysin is supportive of the diagnosis of ONB and excludes a nasal carcinoid (Siudak et al. 2015). The expression of cytokeratin AE1/AE3 highlights the mature neurosensory cells in the olfactory mucosa which naturally express cytokeratin and further supports a neuroepithelial origin. Additionally, the negative staining for cytokeratin 8 allows exclusion of an epithelial tumour of glandular origin. (Kato et al. 2007). Expression of vimentin in ONB has been variably reported(Yamate et al. 2006);(Parker et al. 2010) and in this case was restricted to the tumour stroma. Identification of ultrastructural features of this tumour, including neurite-like processes with microtubular neurofilaments and cytoplasmic, perinuclear electron-dense secretory granules (Martí-García et al. 2023);(Church et al. 2019), would have allowed definitive confirmation. Nonetheless this is not a routine, cost-effective diagnostic test in veterinary oncopathology.

**Table 2:** Immunohistochemical profile and comparison with other nasal neoplasms in goats

Neoplasm	Vimentin	AE1/AE3	CgA	Syp	III beta-tubulin	COX-2	CK8	CD3/ CD20
Olfactory neuroblastoma	+/-	+	-	-	+	-	-	-
Neuroendocrine carcinoma	-	+	+	+	+	+/-	+	-
Adenocarcinoma (ENTV)	-	+	-	-	-	+/-	+	-
Squamous cell carcinoma	-	+	-	-	-	+	+	-
Nasal polyp	-	+	-	-	-	+/-	-	
Lymphoma	+	-	-	-	-	-	-	+

## Data Availability

No datasets were generated or analysed during the current study.
